# Extracorporeal shock-wave therapy in the treatment of pseudoarthrosis: a case report

**DOI:** 10.1186/1757-1626-1-276

**Published:** 2008-10-27

**Authors:** Stefan Endres, Markus Weiskirch, Christiane Hinz, Felix Hütter, Axel Wilke

**Affiliations:** 1 Department of Orthopaedic Surgery, Elisabeth-Klinik, Bigge-Olsberg, Germany

## Abstract

We describe the case of a of a 23 year old man (european) with a complicated fibular-tibial shaft fracture with subsequent pseudoarthrosis formation, which was unable to be corrected by surgery, but which we were finally able to heal using Extracorporeal shock-wave therapy.

## Background

Extracorporeal shock-wave therapy (EWST) has been used for lithotripsy since the 1980s. Haupt was the first to report increased bone growth of the iliac crest after lithotripsy when following patients up. This observation was the first to indicate that shock waves are absorbed by bone and that they elicit a biological response [1].

Subsequent experiments reported trabecular microfractures, periosteal detachment and small haemorrhages which resulted in increased osteoblastic activity and stimulated natural bone healing [1]. Between 1991 and 1998, several working groups reported success rates of between 52 and 89% for the treatment of pseudoarthroses and delayed fracture union with shock waves [2-4].

We describe the case of a complicated fibular-tibial shaft fracture with subsequent pseudoarthrosis formation, which was unable to be corrected by surgery, but which we were finally able to heal using EWST.

## Case presentation

**Figure 1:**
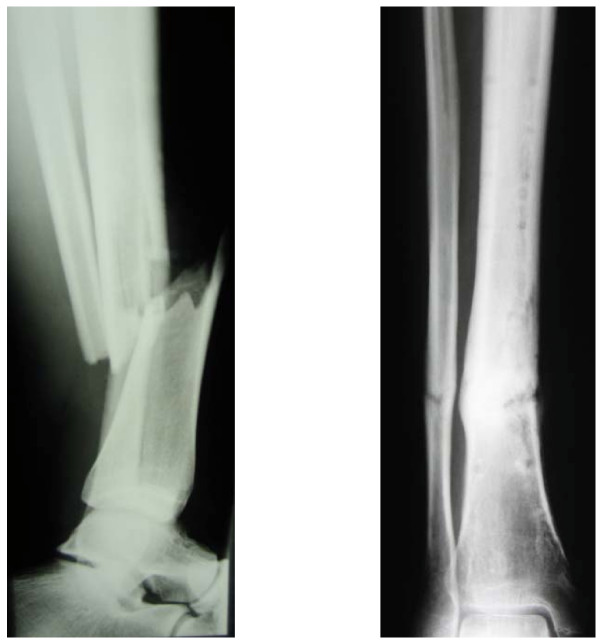
Fracture (left); consolidation of the bone after ESWT (right).

**Figure 2:**
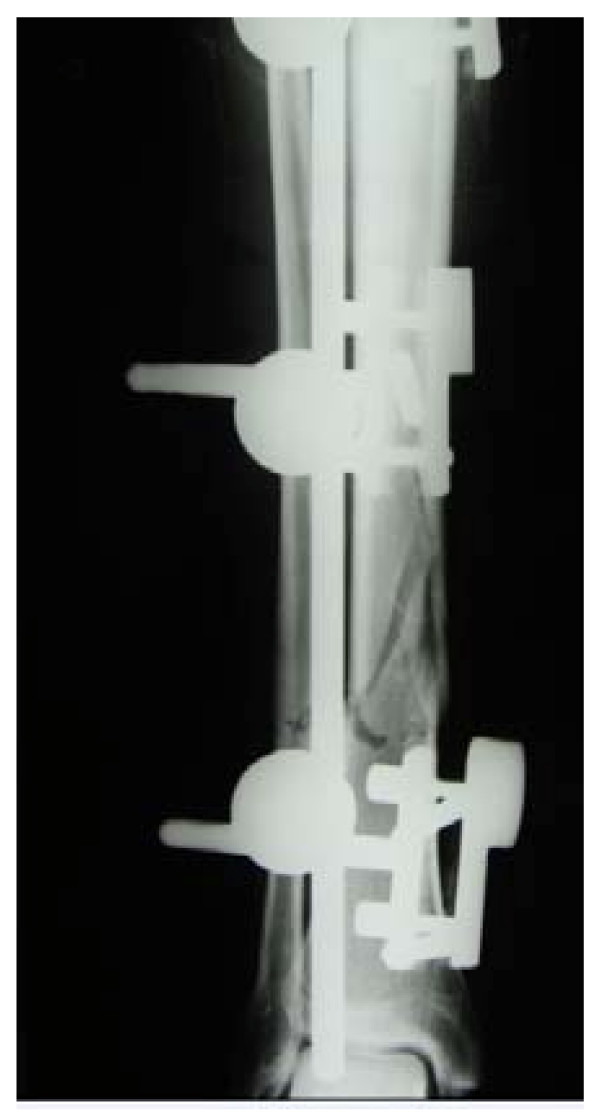
With fixator in place.

A 23-year-old man (european) presented to our emergency department on 30 June 2007 with an open fracture of the lower leg (with distal protrusion) sustained while chopping wood (AO Type A2; Tscherne/Oestern G2 soft tissue injury; Fig. 1). After closed repositioning, an external fixator was applied. The patient was discharged with a normally healed wound and normal circulation and sensory and motor findings on 17 July 2007. (Fig. 2)

The young man remained in our outpatient care. On 13 August 2007, he first noticed weakness when lifting his foot and interdigital dysaesthesia (DI+II). Neurological investigation confirmed partial peroneal paralysis, most likely the result of compartment syndrome. At X-ray, we then established that the fracture gap was not completely ossified with early signs of recurvatum malpositioning and it was decided to perform revision surgery on 5 September 2007.

**Figure 3:**
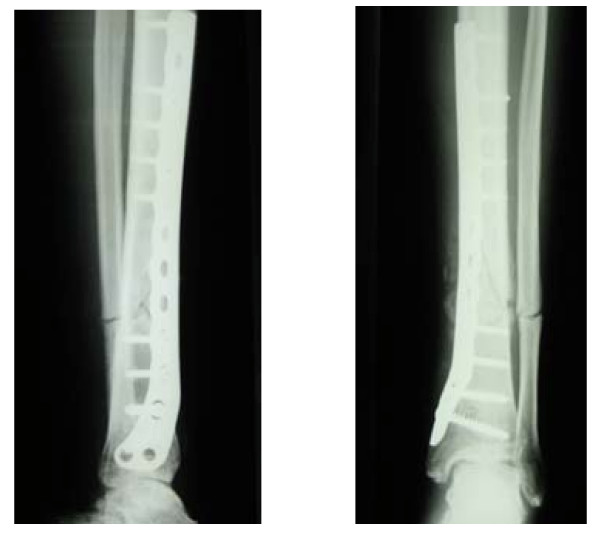
Osteosynthesis plates in place.

Pseudoarthrosis developed (12 weeks postoperative), and it was decided to perform plate osteosynthesis. Surgery for this was conducted on 26 October 2007. (Fig. 3)

**Figure 4:**
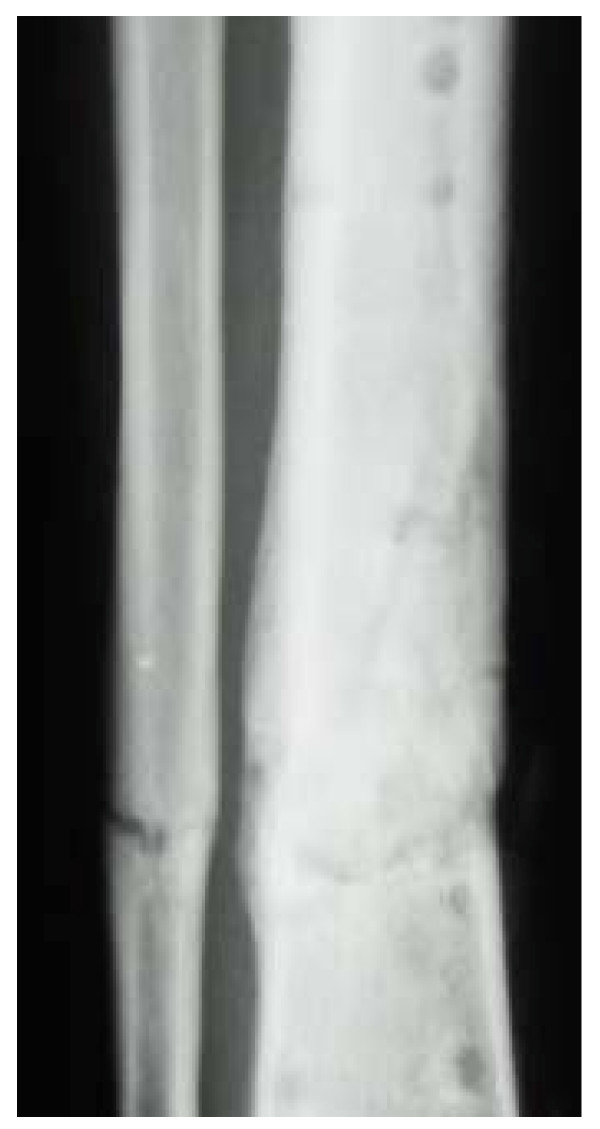
Pseudarthrosis of the tibia and fibula (right).

Callus formation was not observed and the patient developed plate infection, so further revision surgery was conducted. The plates were removed and a Septopal chain was inserted on 13 December 2007. The infection was cured under specific antibiotic therapy and intensive wound management. The infection was finally cleared by further revision surgery and removal of the Septopal chain on 23 January 2008. Wound healing was normal and the peroneal paresis had markedly improved. Although the bone axis was accurate, there was residual structural atrophy of the bone and the fracture gap had still not completely ossified. (Fig. 4)

Based on positive reports on the use of ESWT in the treatment of pseudoarthrosis [2-4], we decided to treat our patient (18 March 2008) with focused shock-wave therapy.

**Figure 5:**
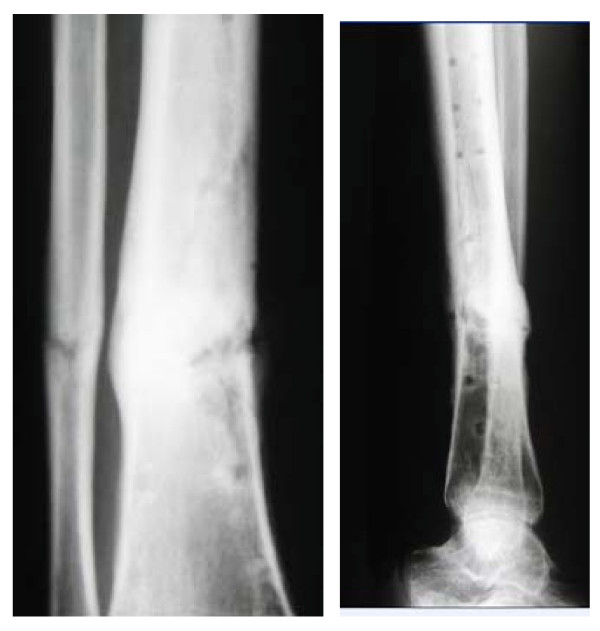
Clear callus formation after EWST.

We used the Duolith SD 1 devcice produced by Storz Medical. We applied 4 treatments with 0.4 mJoule/mm^2^, 3 Hz per session. The sessions were spread over a 6 weeks (1 April-6 May 2008). X-ray follow-up showed a clear increase in the ossification of the fracture gap, and we successfully completed treatment on 27 May 2008. (Fig. 5) The wound was completely normal, the fracture gap was almost completely ossified, the bone axes were accurate, the peroneal paresis had completely subsided, and the patient was completely free from symptoms and was able to walk smoothly.

## Summary

Our case report confirms the efficacy of ESWT and shows that it should be considered when choosing the best approach to the treatment of patients with pseudoarthrosis or delayed fracture union [5].

The largest group of patients treated with this technique were described by Schaden et al., who reported on results in 613 patients. The authors achieved a 76% success rate after a singe ESWT session, and a rate of 56% in patients who needed repeat sessions [5]. It is interesting to note that that success rate was independent of the origin of the pseudarthrosis (hypertrophic, atrophic, or associated with infection).

In the early stages, Schaden et al used high doses of impulses to treat pseudoarthroses (up to 12,000 for long bones). In some cases, however, treatment had to be abandoned at doses of 3,000 to 4,000 impulses for technical reasons. It was, however, established that the low doses of impulses were also adequate to heal pseudoarthroses.

These findings were similar to basic research findings made by Maier et al [2,3], who discovered on murine femurs that the optimum effect of ESWT is reached at an energy level and impulse dosage that causes no histological damage.

Because if these observations, basic research throughout the world began to concentrate more and more on the biological effects of shock waves. Wang et al [4,6,7], Russo et al [8], and Takahashi [9] demonstrated the in vitro release of biologically active substances, such as nitrogen oxide, VEGF, BMP, TGF-1β, IGF-1, and other growth factors [10].

Maier in Germany also showed that shock waves reduce the number of small myelinised nerve cells, which may explain the analgesic effect of shock waves.

Although shock wave therapy cannot replace surgery for the correction of malpositioning, stabilisation of bone fragments or the filling of bone defects, it has a valuable place in the treatment of disturbed bone healing. It has often been stated that prospective, randomized, placebo controlled double-blind studies should be conducted, but these would hardly be practicable in such a complex disorder as pseudoarthrosis, especially because of the problems associated with adequate randomisation.

This leaves only the possibility of case reports or retrospective analyses to enable conclusions to be drawn on the value of ESWT in the treatment of pseudoarthrosis. More and more importance is being attached to the use of ESWT in the treatment of the early stages of osteochondrosis dissecans [11] and necrosis of the head of the femur [12]. ESWT should therefore also be included in the choice of the appropriate treatment for such cases.

## Abbreviations

ESWT: extracorporal Extracorporeal shock-wave therapy; VEGF: vascular endothelial growth factor; BMP: bone morphogenetic protein; TGF-1β: transforming growth factor – 1β; IGF-1: insuline like growth factor – 1.

## Consent

Written informed consent was obtained from the patient for publication of this case report and accompanying images. A copy of the written consent is available for review.

## Competing interests

The authors declare that they have no competing interests.

## Authors' contributions

FH, CH, MW and AW were treating the patient in our hospital until the the patient was completely free from symptoms and was able to walk smoothly. SE was a major contributor in writing the manuscript. All authors read and approved the final manuscript.
